# Does prior acute exercise affect postexercise substrate oxidation in response to a high carbohydrate meal?

**DOI:** 10.1186/1743-7075-5-2

**Published:** 2008-01-27

**Authors:** Wiley Long, Katherine Wells, Virginia Englert, Stacy Schmidt, Matthew S Hickey, Christopher L Melby

**Affiliations:** 1 Department of Food Science and Human Nutrition, and Department of Health and Exercise Science, Colorado State University, Fort Collins, CO 80523, USA

## Abstract

**Background:**

Consumption of a mixed meal increases postprandial carbohydrate utilization and decreases fat oxidation. On the other hand, acute endurance exercise increases fat oxidation and decreases carbohydrate utilization during the post-exercise recovery period. It is possible that the resulting post-exercise increase in circulating nonesterified fatty acids could attenuate the ability of ingested carbohydrate to inhibit lipid oxidation. The purpose of this study was to determine whether prior exercise attenuates the usual meal-induced decline in lipid oxidation.

**Methods:**

Six healthy, physically active young subjects (x age = 26.3 years, 4 males, 2 females) completed three treatments in random order after a ~10 h fast: (a) Exercise/Carbohydrate (Ex/CHO) – subjects completed a bout of exercise at 70% VO_2peak _(targeted net energy cost of 400 kcals), followed by consumption of a carbohydrate-rich meal; (b) Exercise/Placebo (Ex/Placebo) – subjects completed an identical bout of exercise followed by consumption of a placebo; and (c) No Exercise/Carbohydrate (NoEx/CHO) – subjects sat quietly rather than exercising and then consumed the carbohydrate-rich meal. Blood samples were obtained before and during the postprandial period to determine plasma glucose, insulin, and non-esterified fatty acids (NEFA). Respiratory gas exchange measures were used to estimate rates of fat and carbohydrate oxidation.

**Results:**

Plasma NEFA were approximately two-fold higher immediately following the two exercise conditions compared to the no-exercise condition, while meal consumption significantly increased insulin and glucose in both Ex/CHO and NoEx/CHO. NEFA concentrations fell rapidly during the 2-h postprandial period, but remained higher compared to the NoEx/CHO treatment. Carbohydrate oxidation increased rapidly and fat oxidation decreased in response to the meal, with no differences in the rates of carbohydrate and fat oxidation during recovery between the Ex/CHO and NoEx/CHO conditions.

**Conclusion:**

The plasma NEFA concentration is increased during the post exercise period, which is associated with elevated fat oxidation when no meal is consumed. However, when a mixed meal is consumed immediately following exercise, the initially elevated plasma NEFA concentration decreases rapidly, and postexercise fat oxidation during this 2-h postexercise, postprandial period is no higher than that of the 2-h postprandial period without prior exercise.

## Background

Factors that increase fat oxidation can be important in both limiting fat accumulation and increasing loss of body fat stores. Repeated acute changes in fat oxidation resulting from fasting, feeding, and exercise can affect long-term fat balance and body fat stores. During a fasting, post-absorptive state, fatty acid oxidation contributes proportionately more to energy expenditure than does carbohydrate oxidation. This phenomenon is due largely to greater lipid and lower carbohydrate availability, as plasma non-esterified fatty acid (NEFA) concentrations rise in response to lower insulin and higher counter-regulatory hormone concentrations. On the other hand, consumption of a mixed meal (containing carbohydrate, fat and protein) increases blood insulin concentrations which in turn stimulates carbohydrate disposal and increases the contribution of carbohydrate oxidation to energy expenditure while suppressing lipolysis. Fat oxidation, then, decreases in the postprandial state compared to the period prior to meal consumption[1]. The increased availability of postprandial glucose appears to also suppress fatty acid oxidation independent of the anti-lipolytic action of insulin, by inhibiting transport of long-chain fatty acids (LCFA) into the cellular mitochondria[2,3]. Carbohydrate ingestion thus readily increases its own rate of oxidation. Conversely, adding fat to a carbohydrate-rich meal does not increase fat oxidation [1], although experimentally induced elevations of plasma NEFA using infused triacylglycerols can do so [4].

Vigorous acute exercise is characterized by hormonal changes that are opposite that of meal consumption. The rise in catecholamines suppresses pancreatic insulin secretion and increases lipolysis. Despite the increased rate of lipolysis, the mobilization of NEFAs from adipocytes during high intensity exercise may be compromised due to restricted blood flow to adipose tissue [5]. However, during the early recovery period when insulin remains low, catecholamines remain elevated, and with increased blood flow to adipose tissue, plasma NEFA concentrations rise and fat oxidation is elevated [5,6]. It is apparent then, that the hormonal milieu during the post-exercise period favors fat rather than carbohydrate oxidation. We have previously shown fat oxidation to be elevated following both strenuous endurance and resistance exercise [6,7]. Elevations in plasma NEFAs have been shown to compromise glucose transport, as evidenced by infusion of lipids and heparin during hyperinsulinemic, euglycemic clamp studies [8], and it is possible that experimentally raising NEFAs via exercise could produce similar results.

It is not entirely clear what happens when meal consumption, which favors carbohydrate utilization, occurs during exercise recovery, a period which favors fat oxidation. It is possible that the resulting increase in circulating NEFAs could attenuate the ability of ingested carbohydrate to inhibit lipid oxidation. In studies supporting this notion, the contribution of fat oxidation to total energy expenditure was increased during 7-h [9], 10-h [10], and 17-h [11] post-exercise exercise time intervals, which included periods of feeding. For each of these studies, the exercise duration was substantial, lasting 3 hours.

Using a shorter exercise protocol (60 minutes, 50% of VO_2 _max, with a net energy cost of approximately 600 kcal) Dionne et al [12] reported no differences in substrate oxidation over a 24 hour period in which subjects were kept in energy balance, whether they exercised or not. Votruba et al [13], found prior exercise (300 kcal) compared to a no exercise condition produced a sustained increase in oleate oxidation in recovery in response to ingestion of labeled oleate. However, when labeled palmitate was administered, post-exercise palmitate oxidation was not increased relative to the no-exercise trial. It remains unclear what is the effect on substrate oxidation of increasing carbohydrate availability by providing a meal immediately following exercise of the intensity and duration often advocated for health-related fitness (i.e. 60–70% VO_2peak _for 30–60 minutes). Therefore, we determined whether an acute bout of endurance exercise would attenuate the rapid decrease in fat oxidation that normally follows ingestion of a carbohydrate-rich meal. For this study, we used a portion of the data from a previously published study on the effect of prior exercise on the glycemic index [14], and added an additional treatment condition and used the changes in plasma NEFAs and fat oxidation as endpoints, which were not a part of the previous study.

## Methods

### Subjects

Prior to beginning this study, approval from the Human Research Committee at Colorado State University was obtained. The purpose and specific details of the study, as well as possible benefits and risks were discussed with all subjects, both orally and in writing. Subjects were informed that participation in this research was voluntary, and that they could withdraw at any time. Written consent was obtained for all subjects. Subjects were recruited from the campus of Colorado State University. Eight subjects met the enrollment criteria for participation, but one of the subjects failed to complete one of the three treatment conditions required of this study, and instrument failure resulted in missing data for one of the conditions for another subject. Thus complete data were available for six subjects, four males and two females. Subjects were non-smokers, eumenorrheic (females), with no history of eating disorders or chronic health disorders, and with no reported use of any medication which could influence glycemic and insulinemic responses, metabolic rate, and substrate oxidation. None of the participants was obese and all were weight-stable within 2 kg during the previous 6 months. All subjects were regular exercisers, participating in 3 – 5 aerobic exercise sessions per week during the previous 6 months, but none were elite endurance athletes.

### Experimental design

Each subject completed all three of the following treatment conditions, in random order:

**(a) Exercise/Carbohydrate (Ex/CHO)**: completion of an acute bout of exercise at 70% VO_2peak _on a cycle ergometer until a net 400 kcals was expended, followed by consumption of one and a-third servings of a high carbohydrate mixed energy bar (Gatorade^®^, Barrington, IL) containing 50 g of carbohydrate, 6.6 g of fat, and 19.7 g of protein (338 total kcal);

**(b) Exercise/Placebo (Ex/Placebo)**: Same as condition (a), but with subject consuming a placebo (7 kcal as sugar-free Jello, Kraft, Chicago) in place of the bar;

**(c) No Exercise/Carbohydrate (NoEx/CHO)**: Same as condition (a), but with subject sitting quietly for an equivalent period of time instead of exercising.

The female subjects performed each condition between day 7 and 14 of their menstrual cycle in order to remove possible confounding effects of hormonal changes during the cycle that could affect substrate oxidation. Because a 3-day washout period was required between treatments, only 2 conditions could be completed during the 7–14 day period of the monthly cycle. Therefore the female subjects required a longer period of time (i.e. more than one month) to complete all three conditions.

### Specific procedures

#### Preliminary screening

Baseline screenings were conducted to determine eligibility for the study. After providing informed consent, subjects reported to the Nutrition and Metabolic Fitness Laboratory at Colorado State University, where they completed a health history questionnaire, and were screened for self-reported allergies and food intolerances. Body weight and height were measured, a blood pressure reading was taken, and a blood glucose test was performed to rule out any cases of elevated blood pressure or fasting hyperglycemia. None of the female subjects were pregnant.

#### Resting metabolic rate

Resting metabolic rate (RMR) was measured by indirect calorimetry using a CPX Express automated metabolic cart (Medical Graphics, St. Paul, MN). Each subject arrived at the laboratory the morning following a 10-hour overnight fast and before participating in any physical activity. Prior to testing, the metabolic cart was calibrated with known gas concentrations. The subjects were familiarized with the procedure, and then fitted with a nose clip and mouthpiece. As they lay quietly in a semi-recumbent position, resting VO_2 _and VCO_2 _values were collected for 30 minutes, and the final 20 minutes were used for RMR determination. The Weir equation [15] was used to convert gas exchange values into kilocalories expended.

#### Peak oxygen consumption

To determine each subject's sub-maximal exercise intensity during subsequent exercise procedures, peak oxygen consumption was measured using a Monark bicycle ergometer (Stockholm) and a progressive maximal workload protocol. Subjects began with a 2-minute warm-up, pedaling at 80–100 rpm with a zero workload. The workload was then increased approximately 25 watts each minute until the subject could no longer meet the minimum cadence required (20 rpm), or until there was no further increase in oxygen uptake with increased workload. Oxygen uptake and carbon dioxide production were measured using an automated metabolic cart (CPX Express, MedGraphics, St. Paul, MN). Heart rate was monitored during testing using a wireless heart rate monitor (Polar Target™, Hong Kong).

#### Diet standardization

The availability of endogenous carbohydrate (i.e. concentrations of both hepatic and skeletal muscle glycogen) can affect whole-body substrate oxidation rates. Therefore, it was important to control diets for the two days prior to each testing condition to ensure consistent macronutrient intake in order to prevent possible confounding. Subjects were provided with outpatient standardized meals based on measured energy expenditure. Meals were composed of 60% carbohydrate, 25% fat, and 15% protein. For the day two days prior to the testing condition, the caloric content of the meals was equal to 1.5 times the subject's resting metabolic rate (RMR). On the day immediately prior to testing subjects refrained from exercise, so the caloric level was reduced to 1.3 times the RMR. Subjects were instructed to consume all foods provided for the respective days, but in the event all the food could not be eaten, they were to return any uneaten foods. In addition, optional supplemental food modules with the same macronutrient composition were provided, allowing for the consumption of up to 200 additional kcal. This allowed adjustment for the imprecision in determining energy balance over the short period. Ad libitum water intake was allowed both days prior to testing. No food was consumed after 2100 hours the night before testing began the following morning.

#### Experimental testing protocol

Subjects arrived at the Nutrition and Fitness Laboratory at Colorado State University the morning of each testing procedure at 0700 in a fasted state. The subject was weighed, and then pre-exercise O_2 _consumption and CO_2 _production were measured for 15 minutes using indirect calorimetry while the subject sat quietly in a comfortable chair.

##### Exercise protocol

For the exercise conditions, the subject warmed up for five minutes on the Monark bicycle ergometer, reaching an exercise intensity of 70% VO_2peak _at the end of the 5^th ^minute. The subject then continued exercising at 70% of the individual's VO_2peak _for a duration targeted to achieve a net energy cost of ~400 kilocalories. Net kilocalories were calculated by subtracting the RMR from the total kilocalories expended during exercise. Continuous indirect calorimetry using a respiratory face mask was used to measure energy expenditure and also to assure that exercise intensity was maintained at 70% VO_2peak _for the entire exercise period. The respiratory face mask was removed for five minutes every 15 minutes to allow the subject to drink water as needed. The average actual % of VO_2peak _for the Ex/CHO and Ex/Placebo conditions (70.7 ± 1.0 and 71.3 ± 1.4, respectively) only slightly exceeded the objective. Similarly, the net calories expended in the Ex/CHO condition and Ex/Placebo condition (414.8 ± 6.5 and 418.0 ± 4.3, respectively) only slightly exceeded the targeted net exercise energy cost of 400 kcal. For the NoEx condition, the subject rested in a sitting position for an identical amount of time.

##### Blood sampling

Approximately 5 minutes after exercise cessation or non-exercise period in the case of the NoEx/CHO condition, a flexible, indwelling catheter was placed in a superficial forearm vein of the subject. A baseline blood sample was obtained, and the subject then immediately ingested either the placebo or the mixed energy bar within a 5 minute time period. Additional samples of blood were drawn at 15-minute intervals after food or placebo consumption until the end of a 2-hour period, in order to determine glucose, insulin, and NEFA responses over time.

##### Determination of fat oxidation rates

Indirect calorimetry was used to measure energy expenditure and substrate oxidation for the 2-hour period. Subjects lay quietly on a comfortable bed in a semi-recumbent position while VO_2 _and VCO_2 _values were obtained using a respiratory face mask. For purposes of subject comfort, the mask was removed for five minutes of each 15 minute interval during the two hour recovery period and the calculations were then extrapolated to the entire 15 minute interval. Fat oxidation was calculated using VO_2 _and RER data as specified by Jequier et al [16]. First, percentage of energy derived from fat (%E) was calculated using the formula %E = 1-(RER-.707)/(1–.707), which assumes an RER of .707 if only fat is being oxidized. Grams of fat oxidized/minute (g-fat) were calculated using the formula g-fat = (%E)(VO_2_)/1000/2.019. This formula assumes that 2.019 liters of oxygen are consumed per gram of fat oxidized [16].

##### Plasma assays

Blood samples were collected in EDTA tubes, and immediately placed on ice. The tubes were then centrifuged at 2500 rpm for 12 minutes, and plasma was removed and transferred to plastic bullet tubes which were frozen at -70 degrees Celsius until glucose, insulin, and non-esterified fatty acid assays could be completed. Plasma glucose concentrations were determined using the glucose oxidase method via an automated glucose analyzer (YSI 2300 Stat Plus, Yellow Springs Instruments, Inc., Yellow Springs, OH). Plasma insulin and fatty acid concentrations were determined at the Endocrinology Laboratory at the University of Colorado Health Sciences Center using an enzyme-linked immunosorbent sandwich assay for insulin; plasma NEFAs were determined enzymatically.

### Statistical analysis

The data were initially tested for normality and then analyzed using the Statistical Package for the Social Sciences (SPSS, Chicago, IL) software. A within-subjects analysis of variance (ANOVA) was used to compare dependent variables among individuals over time, with statistical significance set at p < 0.05. When time by treatment interactions were significant and condition differences at specific time points were of interest, paired t-tests were used to identify treatment differences during the 2-h post-prandial recovery period.

## Results

### Subject characteristics

The physical characteristics of the subjects are shown in Table 1. All subjects were young, lean, and physically active. There were no significant changes in weight or body mass index (BMI) across the three different trials (not shown in table).

**Table 1:** Physical characteristics of the study participants (n = 6; 4 males, 2 females)

	x ± SEM
Age (years)	26.3 ± 1.4
Weight (kg)	72.35 ± 5.5
Height (cm)	174.6 ± 5.1
BMI* (kg/m^2^)	23.5 ± 1.1
VO_2 _max (L/min)	2.97 ± 0.25
VO_2 _max (ml/kg/min)	41.31 ± 1.49
Max Heart Rate (beats/min)	177 ± 3.8
Max RER^£ ^(VCO_2_/VO_2_)	1.26 ± 0.03

*BMI: Body Mass Index (weight/height^2^).

^£^RER: Respiratory Exchange Ratio

### Acute exercise protocol

Table 2 shows the characteristics of the study subjects during each of the two exercise conditions. There were no between condition differences in any of these characteristics, indicating that the exercise perturbation was similar for the energy bar and placebo conditions.

**Table 2:** Characteristics of the acute exercise protocol (n = 6)

	Ex/Placebo	Ex/CHO
	X ± SEM	X ± SEM
Exercise VO_2 _(% of max)	71.3 ± 1.4	70.7 ± 1.0
Exercise RER* (VCO_2_/VO_2_)	0.97 ± 0.08	0.97 ± 0.02
Exercise time (minutes)	45.6 ± 3.7	45.6 ± 3.7
Total exercise energy cost (kcal)	469.0 ± 5.2	465.9 ± 6.7
Net exercise energy cost (kcal)	418.0 ± 4.3	414.8 ± 6.5

*RER: Respiratory Exchange Ratio

### Glycemic and insulinemic responses

**Figure 1:**
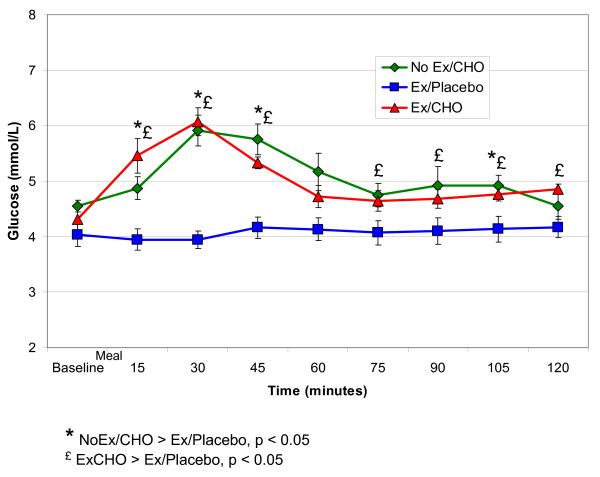
Plasma glucose concentrations (X ± SEM) following exercise or a controlled condition of quiet sitting before (baseline) and during the two hours following ingestion of a high carbohydrate mixed meal bar or a placebo containing 7 kcal as gelatin.

Plasma glucose concentrations are shown for each of the three conditions in Figure 1. Plasma glucose concentrations were higher for both conditions in which carbohydrate was provided (Ex/CHO and NoEx/CHO) compared to the Ex/Placebo condition, with a significant condition by time interaction (p < 0.001). A comparison of only the two carbohydrate conditions (Ex/CHO compared to the NoEx/CHO condition) also revealed a significant condition by time interaction (p < 0.05) owing to a more rapid increase and then decrease in plasma glucose concentrations in the Ex/CHO compared to NoEx/CHO treatments. However, when comparing these two treatment conditions, there was no significant main effect of condition on plasma glucose concentrations.

**Figure 2:**
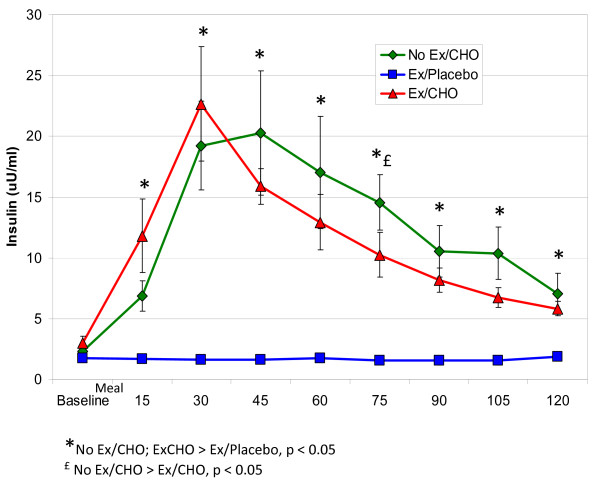
Plasma insulin concentrations (X ± SEM) following exercise or a controlled condition of quiet sitting before (baseline) and during the two hours following ingestion of a high carbohydrate mixed meal bar or the placebo.

Plasma insulin concentrations are shown for each of the three conditions in Figure 2. Insulin concentration increased rapidly for the Ex/CHO condition, peaking at 30 minutes at which time it decreased sharply. Given that the insulin concentration did not change over time for the placebo condition, when the Ex/Placebo condition was compared to the Ex/CHO condition, there was a time by condition interaction (p < 0.001). A comparison of the NoEx/CHO condition to the Ex/CHO condition showed a condition by time interaction as well (p = 0.03); the insulin concentration for the Ex/CHO condition rose faster than the NoEx/CHO condition for the first 30 minutes during the post-prandial period, but then fell more rapidly during the last 90 minutes of this 2-h period. There was no significant main effect of condition on plasma insulin when comparing the Ex/CHO and NoEx/CHO treatments.

### Plasma [NEFA]

**Figure 3:**
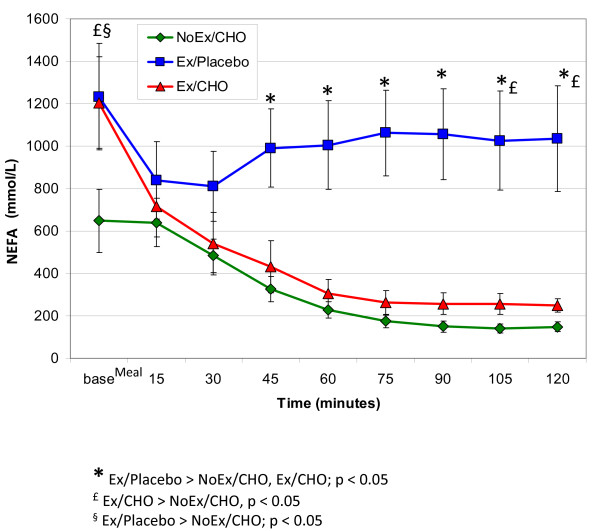
Plasma nonesterified fatty acids (NEFAs) (X ± SEM) following exercise or a controlled condition of quiet sitting before (baseline) and during the two hours following ingestion of a high carbohydrate mixed meal bar or the placebo.

Figure 3 shows plasma NEFA concentration for the two hour post-exercise period, including an initial baseline measurement taken at time zero, prior to consumption of the placebo or meal. The interaction and main effects of condition and time were all statistically significant a p < 0.05. Immediately post-exercise, plasma NEFA concentrations were nearly twice as high for the exercise conditions compared to the no exercise condition. Concentrations remained significantly higher for the Ex/Placebo condition compared to NoEx/CHO and Ex/CHO for the last 75 minutes of the 2-h measurement period. Following meal consumption, NEFA concentrations fell rapidly for both Ex/CHO and NoEx/CHO and were not significantly different from each other during the first 45 minutes after baseline. However, during the second hour of the postprandial period, NEFA concentrations were modestly higher for Ex/CHO compared to NoEx/CHO, with the difference reaching statistical significance during the final 30 minutes (p < 0.05).

### Recovery RER

**Figure 4:**
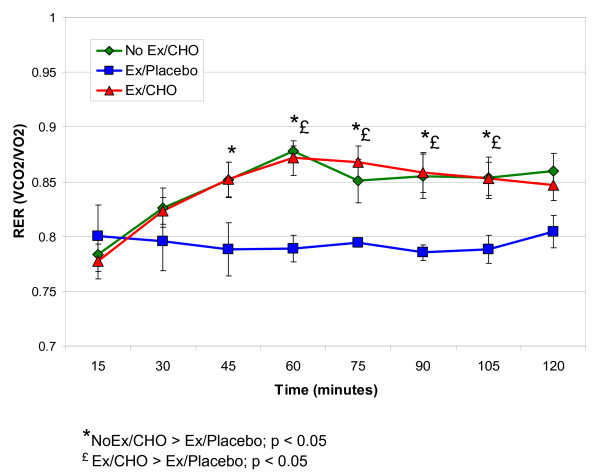
Respiratory exchange ratio values (VCO_2_/VO_2_) (X ± SEM) after exercise or a controlled condition of quiet sitting and following ingestion of a mixed meal bar or the placebo.

The respiratory exchange ratios (RER) are shown for each of the three conditions in Figure 4. When the conditions were compared, there was a time by condition interaction (p = 0.005), with pairwise comparisons indicating that the RER values for both carbohydrate conditions compared to the placebo condition were significantly higher during most of the 15-min time intervals of the 2-h postprandial recovery period. The RER values for the NoEx/CHO condition and the Ex/CHO condition were not significantly different, indicating that there were no differences between these two treatments in the relative proportions of oxygen used for fat and carbohydrate oxidation, respectively.

### Fat oxidation

**Figure 5:**
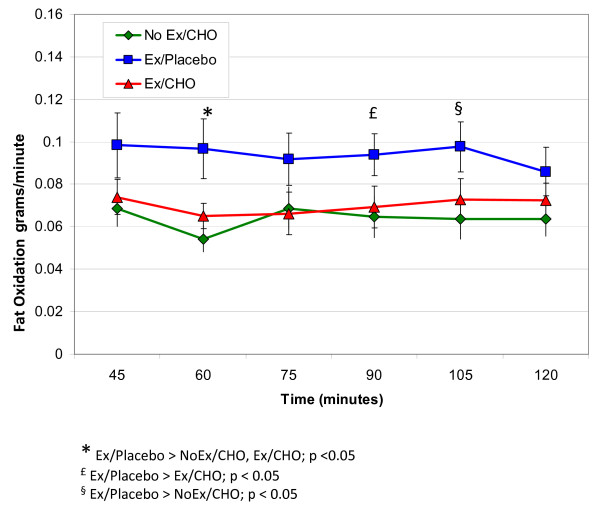
Calculated rates of fat oxidation (g/min averaged over 15 minute intervals) (X ± SEM) based on indirect calorimetry during the final 90 minutes of the 120 period following ingestion of the meal bar or the placebo.

The RER values by themselves only provide information about the relative and not absolute contributions of macronutrient oxidation to energy expenditure. Also, at a given RER, the absolute rate of fat oxidation will be greater with higher rates of oxygen consumption. Therefore, given the higher oxygen consumption during recovery from exercise compared to the no-exercise condition, we calculated the absolute rates of fat oxidation from the RER and VO_2 _values during the recovery period. Because of potential disturbances in the bicarbonate ion pool during the early portion of the recovery period which precludes accurate calculation of substrate oxidation rates from respiratory gas exchange measures, fat oxidation was not calculated during the first 30 minutes. The average oxygen consumption during the final 90 minutes of the 2-h measurement period was significantly higher for ExCHO (x ± SE = 295 ± 24 ml/min) compared to ExPlacebo (262 ± 28) and NoExCHO (272 ± 22). Figure 5 shows the absolute rate of fat oxidation (g*min^-1^) calculated from RER and VO_2 _values over the last 90 minutes of the post-exercise postprandial period. Among the two exercise conditions, the rate of fat oxidation was reduced by provision of the carbohydrate-rich energy bar compared to placebo (p for treatment by time interaction <0.001). Post hoc analyses at specific time points revealed that fat oxidation was significantly higher in the Ex/Placebo condition when compared to the Ex/CHO condition at 60 and 90 minutes (p <0.05). The 75-minute (p = 0.06) and 105-minute (p = 0.08) time points approached, but did not reach statistical significance. There was no difference in fat oxidation between the Ex/CHO condition and the NoEx/CHO condition at any time points during the 90-min postexercise, postprandial period (all p-values > 0.5).

## Discussion

The purpose of this study was to determine whether the elevation of plasma NEFA concentrations that occurs during recovery from acute endurance exercise would attenuate the decrease in fat oxidation in response to ingesting a carbohydrate-rich mixed meal during the recovery period. The major finding was that despite two-fold increases in NEFAs during the early post-exercise period, a small carbohydrate-rich mixed meal consumed immediately after exercise resulted in rapid decreases in plasma NEFA concentrations and a rapid shift in fuel utilization toward greater carbohydrate and less fat oxidation. Thus, in regard to macronutrient partitioning, ingestion of a high carbohydrate meal with concomitant elevation of plasma insulin appears to readily override the impact of initially elevated NEFA, resulting in a rapid decrease in both NEFA and the rate of whole-body fat oxidation.

### Macronutrient metabolism

Plasma glucose and insulin concentrations rose rapidly during the first 30 minutes following meal ingestion, whether preceded by exercise (Ex/CHO) or not (NoEx/CHO). There was a steeper rise in glucose for the first 30 minutes in the ExCHO condition followed by a more rapid fall. This may be explained by an increased rate of gut glucose absorption following exercise. Rose et al [17] found that in comparison to a no-exercise condition, a 75 g oral glucose load given 30 minutes after a 55 minute exercise session at 70% of VO_2peak _resulted in a 25% greater rate of gut glucose absorption. This suggests that post-exercise adaptations in splanchnic tissues facilitate a greater rate of glucose appearance following glucose ingestion. The increases in plasma glucose and insulin immediately following meal ingestion would increase glucose transport, making glucose readily available for both storage and oxidation in skeletal muscle.

Plasma NEFA concentrations were significantly elevated following both exercise conditions prior to meal ingestion. The increased availability of plasma NEFA during exercise recovery has been shown to increase skeletal muscle and whole-body fatty acid oxidation [18]. In the Ex/Placebo condition, which is essentially exercise and fasting, plasma NEFA remained elevated for most of the 2-h post-exercise recovery period and was associated with significantly lower RER values, reflecting higher rates of fat oxidation. Elevated NEFAs have been shown to interfere with glucose transport, resulting in less carbohydrate available for oxidation [8], hence our hypothesis that prior exercise would enhance post-prandial fat oxidation. Intense exercise lowers skeletal muscle and hepatic glycogen, and thus carbohydrate ingested during recovery could favor glycogen restoration over oxidation. The ingestion of the carbohydrate-rich meal rapidly lowered plasma NEFA in accordance with the sharp rise in insulin known to inhibit lipolysis and promote fatty acid re-esterification and storage as triacylglycerols, although during the last 30 minutes of the recovery period NEFA remained significantly higher for Ex/CHO compared to NoEx/CHO. However this modest elevation may have little physiologic relevance as it was not associated with a corresponding elevation in fat oxidation.

In the present study, the failure of prior acute exercise to attenuate increases in postprandial RER seems at odds with previous findings. Note however, that most of the studies [9-11] that found exercise to lower postprandial RER (i.e. higher proportion of fat used as a fuel) have used long duration exercise bouts (i.e. 3 hours) and examined post-exercise periods ranging from 7 to 24 hours, with meal consumption not occurring immediately after cessation of exercise. It is possible that prior acute exercise lowers RER in the postexercise, postprandial state only when the exercise perturbation is of adequate duration to create a substantial acute energy deficit, and when there is a significant time interval between exercise cessation and meal consumption (i.e. an hour or two). The additional time prior to meal consumption would result in a longer period of elevated plasma NEFAs and greater cellular uptake of fatty acids, a milieu that would appear more favorable to lipid oxidation and subsequent compromised postprandial glucose transport and oxidation. Experimental studies suggest that an extended period of elevated plasma NEFAs is required to attenuate glucose transport. Itani et al [8] found that experimentally raising blood NEFA concentrations via lipid-heparin infusions for 6 hours caused significant reductions in insulin stimulated glucose transport during the final 4 hours, but had no effect during the initial 2 hours. In our study, with meal consumption immediately following exercise cessation, the modestly higher NEFA concentration following meal ingestion during exercise recovery appears to be insufficient to attenuate glucose transport and enhance fat oxidation.

### Strengths and caveats

Energy balance and macronutrient composition of the diet can influence substrate oxidation rates. We therefore controlled exercise energy expenditure and standardized energy and macronutrient intake during the two days prior to each subject completing the three separate treatments. Also, because menstrual cycle phase may influence post-exercise substrate oxidation rates [19], each female subject completed all three treatment protocols during the same phase of the cycle.

Despite the small sample size in our study, using a within-subjects design was more than adequate to demonstrate the significant effect of exercise and meal consumption on NEFA changes. Marion-Latard et al [18] showed a significantly elevated rate of fat oxidation following exercise compared to no-exercise using the same sample size as our study. Given the lack of any evidence demonstrating differences in postprandial RER and rates of fat oxidation between exercise and no exercise conditions (Ex/CHO: RER = 0.844, SE = 0.012; NoEx/CHO: RER = 0.845, SE = 0.014, p = 0.94; Ex/CHO: g fat/min = 0.070, SE = 0.003; NoEx/CHO: g fat/min = 0.064, SE= 0.007, p = 0.44), it is improbable that a larger sample size would change our findings.

Several potential caveats should be noted. It is possible that gender differences could occur in response to the exercise and dietary perturbations. However, because each subject served as his/her own control, our results are not likely confounded by gender. We did not measure urinary nitrogen during the study, thus we can provide no information regarding the contribution of protein to energy expenditure during the 2-h post-exercise/control period. However, it is generally recognized that amino acid oxidation is a minor contributor to overall energy expenditure during exercise and post-exercise and in response to a small mixed meal. Also, the measurement of urinary N during such a short time interval (i.e. exercise and 2-h following) will not provide accurate estimates of amino acid oxidation during this same time period, owing to exercise induced changes in urine output and time lags between amino acid oxidation and appearance of N in the urine.

## Conclusion

In summary, our results demonstrate that exercise of the duration and intensity used for health-related fitness results in significant elevations in post-exercise plasma NEFAs. However, when a carbohydrate-rich mixed meal is consumed immediately following cessation of exercise, the plasma NEFA concentrations fall dramatically, carbohydrate oxidation increases, and there is no attenuation of meal induced declines in fat oxidation. Thus, with a modest exercise perturbation, ingestion of a mixed meal with attendant elevations of plasma insulin and glucose overrides the impact of initially elevated fatty acids. The effects on fat oxidation of different time intervals between exercise and meal consumption should be studied further.

## Competing interests

CM has received speaking honoraria from Gatorade, Inc. All other authors declare that they have no competing interests.

## Authors' contributions

WL helped design the study, test the subjects, analyze the data, and write the manuscript. KW helped design the study, test the subjects, and contributed to the data analysis. VE helped design the study, coordinated the subject testing, and helped with data analysis. SS helped test the subjects and write the manuscript. MSH helped with the study design, supervision of subject testing, and manuscript writing. CLM conceived of the study, supervised subject testing, coordinated the blood assays, supervised the data analyses, and helped write the manuscript.
